# BRAD V3.0: an upgraded Brassicaceae database

**DOI:** 10.1093/nar/gkab1057

**Published:** 2021-11-10

**Authors:** Haixu Chen, Tianpeng Wang, Xiaoning He, Xu Cai, Runmao Lin, Jianli Liang, Jian Wu, Graham King, Xiaowu Wang

**Affiliations:** Institute of Vegetables and Flowers, Chinese Academy of Agricultural Sciences, No.12, Haidian District, Beijing 100081, China; Institute of Vegetables and Flowers, Chinese Academy of Agricultural Sciences, No.12, Haidian District, Beijing 100081, China; Institute of Vegetables and Flowers, Chinese Academy of Agricultural Sciences, No.12, Haidian District, Beijing 100081, China; Institute of Vegetables and Flowers, Chinese Academy of Agricultural Sciences, No.12, Haidian District, Beijing 100081, China; Institute of Vegetables and Flowers, Chinese Academy of Agricultural Sciences, No.12, Haidian District, Beijing 100081, China; Institute of Vegetables and Flowers, Chinese Academy of Agricultural Sciences, No.12, Haidian District, Beijing 100081, China; Institute of Vegetables and Flowers, Chinese Academy of Agricultural Sciences, No.12, Haidian District, Beijing 100081, China; Southern Cross Plant Science, Southern Cross University, Lismore, New South Wales, Australia; Institute of Vegetables and Flowers, Chinese Academy of Agricultural Sciences, No.12, Haidian District, Beijing 100081, China

## Abstract

The Brassicaceae Database (BRAD version 3.0, BRAD V3.0; http://brassicadb.cn) has evolved from the former *Brassica* Database (BRAD V2.0), and represents an important community portal hosting genome information for multiple *Brassica* and related Brassicaceae plant species. Since the last update in 2015, the complex genomes of numerous Brassicaceae species have been decoded, accompanied by many omics datasets. To provide an up-to-date service, we report here a major upgrade of the portal. The Model-View-ViewModel (MVVM) framework of BRAD has been re-engineered to enable easy and sustainable maintenance of the database. The collection of genomes has been increased to 26 species, along with optimization of the user interface. Features of the previous version have been retained, with additional new tools for exploring syntenic genes, gene expression and variation data. In the ‘Syntenic Gene @ Subgenome’ module, we added features to view the sequence alignment and phylogenetic relationships of syntenic genes. New modules include ‘MicroSynteny’ for viewing synteny of selected fragment pairs, and ‘Polymorph’ for retrieval of variation data. The updated BRAD provides a substantial expansion of genomic data and a comprehensive improvement of the service available to the Brassicaceae research community.

## INTRODUCTION

BRAD is a genome database and analysis portal that has been available to the *Brassica* research community for over a decade since its first release in 2010 (1). It represents a major resource for understanding complex genome structures and their association with evolutionary and domestication history. The Brassicaceae is a large family of flowering plants with 4636 known species in 340 genera (2). It includes many crops of agricultural, ornamental, condimental, medicinal and scientific significance (3), as well as the model plant species *Arabidopsis thaliana* for which extensive gene functional information has been accumulated over the past 30 years. BRAD was the first comprehensive database hosting whole genome information for the first sequenced *Brassica* species, *B. rapa*, a mesohexaploid diploid resulting in a chromosomally segmented triplicated genome (4). The original key feature of BRAD was to associate the functional information for *A. thaliana* genes annotated in TAIR (5) with those identified in a *Brassica* crop through their syntenic relationships at the subgenome level. The subsequent sequencing of additional Brassicaceae genomes enabled detailed syntenic analysis to be extended to include 13 Brassicaceae species within BRAD v2.0 in 2015 (6).

Due to the rapid advances of sequencing technology in recent years, high-quality reference genome sequences of many Brassicaceae species have been either decoded or upgraded (7–35). This includes revisions to reference *B. rapa* (V3.0), *B. oleracea* (V2.0) and *B. nigra* (V2.0) as well as *de novo* assemblies for *Brassica carinata*, *Isatis indigotica* and *Raphanus sativus* and several other species. Several independent online genome databases and portals have been developed to host *Brassica* genome information. The www.brassica.info site is an information and reference portal for the Multinational Brassica Genome Project. In addition, the following provide specific browsers or analysis tools: the *Brassica napus* pan-genome information resource, BnPIR (36); SNP Database of *Brassica napus*, BnaSNPDB (37); The *Brassica oleracea* Genome Database, Bolbase (38); A Gene Expression Database for *Brassica* Crops, BrassicaEDB (39); The Genomic Variation Database of *B. napus*, BnaGVD (40). These databases have different capabilities, although for each their scope is limited either to a specific species, or a specific data type. BRAD remains the only genome database and analysis platform that can provide information linked across a number of Brassicaceae species with a wealth of gene and genomic features.

The previous version of BRAD was built to provide a service for a fixed number of genomes. In consideration of the rapid increase of genomic data volume and updating speed, we rebuilt BRAD using the Model–View–ViewModel (MVVM) framework. The site has moved from the ‘http://brassicadb.org’ domain hosted on an institutional server to the ‘http://brassicadb.cn’ domain hosted in a cloud server.

## MATERIALS AND METHODS

**Figure 1.**
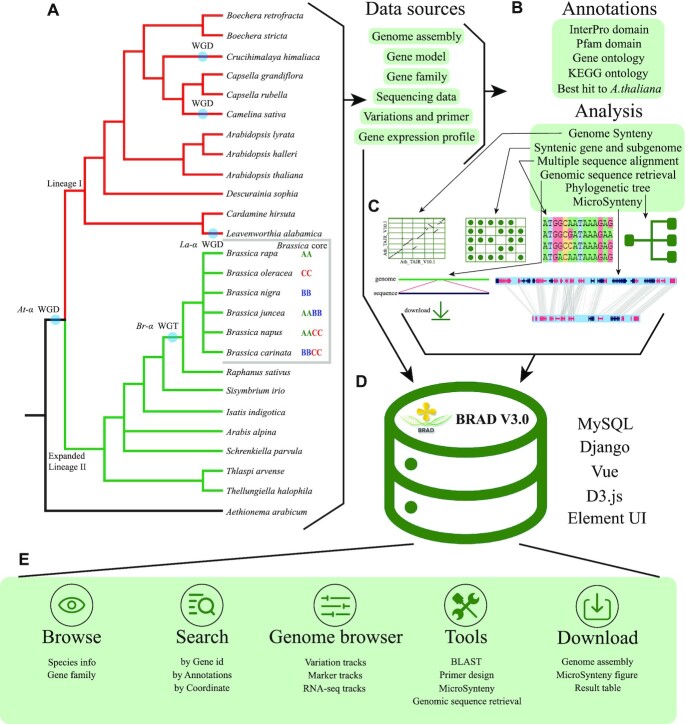
Overview of the Brassicaceae Database, BRAD. (**A**) Data sources included 25 species, 35 reference genomes in Brassicaceae and associated gene models, RNA-seq data. Lineage I and Expanded Lineage II were originally described by Mandakova et al (65–68). (**B**) Summary of data processing methods. (**C**) Display of web tools. (**D**) All data is stored in the MySQL relational database. The Django and Vue framework are used for interactive queries between the front and back ends. (**E**) An overview of the Web interface and the usage of the BRAD.

All data sources, data processing, web interface, and features of BRAD V3.0 are summarized in Figure 1. It consists of five parts: data collection, gene functional annotation, online analysis and visualization, database construction, web interface and toolkit development. The programs and packages used in the process are summarized in Supplementary Table S1.

### Data sources

Based on data from previous version, when identifying genomic data to be included within BRAD, we wished to represent evolutionarily or economically important species of Brassicaceae that met the following criteria: being hosted in the version 2.0, of either having an upgraded assembly version, or that the assembly for a new Brassicaceae species was of high quality. Resequencing data for *B. rapa*, *B. oleracea* and *B. napus* were from published papers (41–43). All RNA-seq data were downloaded from NCBI SRA database (www.ncbi.nlm.nih.gov/sra) to represent as many organs or tissues as possible for each species, the metadata for which are summarised in Supplementary Table S2.

### Syntenic Gene @ Subgenome

BLAST (44) was used by SynOrths (45) for the identification of syntenic genes. We reperformed syntenic gene analysis across all genomes in order to ensure consistency for synteny identification. In total, pairwise syntenic genes were identified from all protein-coding genes based on *A. thaliana* and *B. rapa* V1.5 using the default parameters of SynOrths. For species that have not undergone a *Brassica* whole-genome triplication (*Br-α* WGT) event, synteny was detected in relation to *A. thaliana*. For species with a *Br-α* WGT event (Figure 1A), three sets of subgenomes were allocated based on the *B. rapa* genome (Chiifu) V1.5, and then the genes in each subgenome were compared to the *A. thaliana* gene set. For *Camelina sativa*, we used the available subgenome information from supplementary data (20). For *Leavenworthia alabamica*, we treated it as a species that has not undergone WGT because its genome is of poor quality (Supplementary Figure S2). However, because *A. thaliana* is not the direct *Brassiceae* ancestor having a tPCK karyotype (46), species with a *Br-α* WGT event will lose syntenic gene pairs that otherwise are unique in the tPCK karyotype species. Therefore, the latter species were further compared with *B. rapa* genome V1.5, so that genes lacking synteny with *A. thaliana* may form syntenic gene pairs with *B. rapa* genome V1.5. Based on the synteny table for *A. thaliana* and *B. rapa* (46), we integrated these syntenic gene pairs into a combined synteny table (Supplementary Figure S1A).

### Multiple sequence alignment and gene phylogeny

The available gene, CDS and protein sequences integrated in the BRAD-MySQL database are available for online analysis. After receiving user query parameters from the browser, the BRAD server queries the corresponding sequences in the database and writes it to a .fasta file. This file is then used to compute multiple sequence alignment and phylogenetic tree results using the default parameters of MUSCLE (47,48). Sequence and alignment results are packaged in JSON format and sent to the browser, with multiple sequence alignment rendered using the Vue plugin vue-svg-msa and phylogenetic tree results using vue-phylogram.

### Polymorph

We added genomic variations for 1251 re-sequenced genotype accessions, including two sets for *B. rapa*, where one represented 524 accessions and used *B. rapa* (Chiifu) V3.0 as the reference genome (41), and the other was a subset of 199 from 524 accessions used (Chiifu) V1.5 as reference (42). Two *B. oleracea* data sets representing 119 diverse accessions used *B. oleracea* (JZS) V1.1 (42) and (JZS) V2.0 as the reference genomes. The *B. napus* data set represented 608 diverse accessions (43) and used the (Darmor-bzh) V5 genome as the reference. We further filtered the variation data with MAF ≥0.05 and missing rate ≤0.1, and built a phylogenetic tree using the default parameters of PHYLIP (49). This generated a tree file that was rendered with iTOL (50). The corresponding data could be retrieved from the database, with SVG images rendered on the web page.

### Gene function and functional elements annotation

We used the gene models as described in the original publications for each of the genomes, but re-annotated all of them (Figure 1B). The protein sequences were further compared against the InterPro database (51) using the default parameters of InterProScan (52) to identify InterPro domains, Pfam domains (53) and gene ontology (54). The KEGG orthology (55) was annotated using the KAAS web server (56). All genes except those from *A. thaliana* have been compared with *A. thaliana* using BLASTP, where the parameters of BLASTP alignment to be satisfied included: identity >70%, coverage of query gene >75%, coverage of subject gene >75%. The gene with the highest identity was defined as the BLASTX (best hit) match to *A. thaliana*.

### RNA-seq data analysis

HISAT2 (57) was used to map reads to the respective reference genome for each species. Samtools (58) was used to generate the sorting bam files. FeatureCounts (59) was used to calculate the number of reads. The TPM (transcripts per million) values and standard deviations were calculated using custom Python script. For all software mentioned are default parameters were used.

### Genomic sequence retrieval and MicroSynteny

In the ‘Genomic sequence retrieval’, the Biopython (60) was used within a custom Python script to read the genome sequence and serve the genomic sequence to users. Based on ‘Genomic sequence retrieval’, we developed the ‘MicroSynteny’ module. Firstly, this obtains the sequences after receiving the query data transmitted by the web page. Secondly, the second sequence is used to build the BLAST library, and the first sequence acts as the query sequence. Finally, the collated results, including the gene and CDS information in the two fragments extracted from the database, are packaged in JSON format and sent to the browser and rendered by D3.js (https://d3js.org/). The methodology for this tool is described in detail in Supplementary Figure S1B.

### Data integration

Syntenic genes, non-syntenic ortholog genes, transcriptome data, genomic synteny data and gene annotations were integrated in the BRAD-MySQL database. For variation data, we used the method of separating samples and data for storage and reading, and custom Python scripts for integration in the background. The relationships between data sets and main functions is shown in Supplementary Figure S1C.

### Database construction and Web interface

All pre-processed data were integrated into the BRAD-MySQL database (Figure 1D). It contains a table of all gene model information, a table of all syntenic genes and a table of subgenome information, as well as a table including gene sequence, CDS sequence and protein sequence. The Django framework was used to query data from the database backend and build an API interface to send the data obtained from database to the browser. The Vue framework was used to develop a user-friendly web interface, and to implement multiple custom dynamic charts using the Element UI library.

### Toolkit development

Primer3 (61) was used to design primers for specific parameters. Synteny viewer was developed to visualize the synteny between different genomes. Web BLAST was driven by Sequenceserver (62). Genome sequences, gene models, markers, variation and omics data were displayed by JBrowse (63).

## RESULTS

### Overview of data

The BRAD covers a comprehensive representation of species types from the Brassicaceae family. V3.0 includes 36 genome assemblies of 26 species from 12 tribes (64) within the Brassicaceae (Figure 1A, Table 1, Supplementary Table S3). Compared with V2.0, 23 additional genome assemblies were included representing 13 species and 6 tribes. Thirteen genome assemblies were from Lineage I, 22 from Expanded Lineage II, and *Aethionema arabicum* represent an ancestral species (65–68). At the species level, 12 are from Lineage I and 13 from Expanded Lineage II. At the tribe level, five are from Lineage I and six from Expanded Lineage II. In addition, BRAD v3.0 also provides different versions of the genomes for the economically important *Brassica* crop species, which will assist in a wide range of studies.

**Table 1.** Genomic information included in the database

Abbreviation	Species	Genome assembly	Pub. date	Contig N50	Scaffold N50	Genome size (Mb)	Source
Araal_Paj_V4	*Arabis alpina*	version 4	02 Feb 2015	-	788 kb	375	(22)
Aetar_V1.0	*Aethionema arabicum*	version 1.0	30 Jun 2013	-	118 kb	191	(10)
Araha_Tad_V1.1	*Arabidopsis halleri*	version 1.1	27 Sep 2016	-	712 kb	124	(21)
Araly_MN47_V1.0	*Arabidopsis lyrata*	version 1.0	10 Apr 2011	-	-	200	(8)
Araly_MN47_V2.1	*Arabidopsis lyrata*	version 2.1	-	-	-	201	phytozome V12
Ath_TAIR_V10.1	*Arabidopsis thaliana*	version 10.1	14 Dec 2000	-	-	116	(7)
Braca_zd1_V1.0	*Brassica caritana*	Version 1.0	04 Feb 2021	1.44 Mb	60 Mb	1113	(11)
Braju_tum_V1.5	*Brassica juncea*	version 1.5	05 Sep 2016	61 kb	855 kb	784	(25)
Brana_ZS_V2.0	*Brassica napus*	version 2.0	28 Aug 2017	39.57 kb	602.22 kb	932	(28)
Brana_Dar_V5	*Brassica napus*	version 5	04 Sep 2014	-	-	850	(34)
Brana_Dar_V10	*Brassica napus*	version 10	15 Dec 2020	11.48 Mb	-	896	(33)
Brana_ZS_PB_V1.0	*Brassica napus*	version PB 1.0	19 Oct 2020	1.64 Mb	-	888	(27)
Brani_San_V1.1	*Brassica nigra*	version 1.1	30 Jun 2020	1.48 Mb	68.5 Mb	388	(29)
Brani_Ni100_V2	*Brassica nigra*	version 2.0	03 Feb 2020	17.1 Mb	-	491	(26)
Boere_V1.0	*Boechera retrofracta*	version 1.0	28 Mar 2018	-	2.3 Mb	217	(9)
Boest_V1.2	*Boechera stricta*	version 1.2	-	-	-	184	phytozome V12
Braol_JZS_V1.1	*Brassica oleracea*	version 1.1	23 May 2014	26 kb	1.45 Mb	374	(12)
Braol_JZS_V2.0	*Brassica oleracea*	version 2.0	09 Aug 2020	2.37 Mb	-	542	(30)
Braol_HDEM_V1.0	*Brassica oleracea*	version 1.0	02 Nov 2018	9.49 Mb	29.52 Mb	539	(16)
Brara_Chiifu_V1.5	*Brassica rapa*	version 1.5	28 Aug 2011	27 kb	1.97 Mb	279	(4)
Brara_Chiifu_V2.5	*Brassica rapa*	version 2.5	03 Apr 2017	-	3.38 Mb	375	(13)
Brara_Chiifu_V3.0	*Brassica rapa*	version 3.0	15 Aug 2018	1.45 Mb	4.44 Mb	341	(31)
Brara_Z1_V1	*Brassica rapa*	version 1.0	02 Nov 2018	5.52 Mb	15.39 Mb	390	(16)
Carhi_V1.0	*Cardamine hirsuta*	version 1.0	31 Oct 2016	-	509.5 kb	193	(15)
Capgr_V1.1	*Capsella grandiflora*	version 1.1	-	-	-	103	phytozome V12
Cruhi_V1.1	*Crucihimalaya himalaica*	version 1.1	20 Mar 2019	-	2.09 Mb	427	(23)
Capru_Mon_V1.1	*Capsella rubella*	version 1.1	09 Jun 2013	134.1 kb	15.1 Mb	135	(14)
Camsa_DH55_V1.0	*Camelina sativa*	version 1.0	23 Apr 2014	33.41 Kb	30.09 Mb	619	(20)
Desso_V1.1	*Descurainia sophioides*	version 1.1	-	-	-	124	phytozome V12
Isain_V1	*Isatis indigotica*	version 1.0	01 Feb 2020	1.18 Mb	36.17 Mb	294	(17)
Leaal_V1.0	*Leavenworthia alabamica*	version 1.0	30 June 2013	-	70 kb	167	(10)
Rapsa_Xiang_V1.0	*Raphanus sativus*	version 1.0	01 Nov 2015	39.62 kb	1.35 Mb	381	(18)
Sisir_V1.0	*Sisymbrium irio*	version 1.0	30 June 2013	-	135 kb	249	(10)
Schpa_V1.0	*Schrenkiella parvula*	version 1.0	07 Aug 2011	5.29 Mb	-	134	(24)
Thlar_MN106_V1.1	*Thlaspi arvense*	version 1.1	27 Jan 2015	140.82 kb	-	406	(19)
Theha_V1.0	*Thellungiella halophila*	version 1.0	21 Mar 2013	272 kb	8 Mb	235	(35)

The syntenic genes identified differed slightly from BRAD V2.0 (Supplementary Table S4). More than 1 million syntenic genes were identified from all protein-coding genes, among which 1 052 853 were syntenic with *A. thaliana* genes. Of all species except *A. thaliana*, 11 were derived from Lineage I, of which *Crucihimalaya himalaica*, *Camelina sativa* and *Leavenworthia alabamica* have been reported as having undergone WGT. Among these, we identified 27 225, 57 423 and 16 836 syntenic genes respectively. The low number of syntenic genes in *Leavenworthia alabamica* was largely due to the low quality of genome assembly. In the remaining eight species, we identified 17 591–21 132 syntenic genes. There are 13 species belong to Expanded Lineage II. For the four diploid *Brassiceae* species, we collected four assemblies of *B. rapa*, three of *B. oleracea*, two of *B. nigra* and one *R. sativus*. Among them, we identified 25 173–34 578 syntenic genes. For the three allotetraplloid *Brassiceae* species, we collected four assemblies of *B. napus*, one of *B. carinata* and one of *B. juncea*. Among them, we identified 49 442–66 872 syntenic genes. In the remaining six species that have not undergone WGT, we identified 12 968–19 016 syntenic genes. Besides, we identified 91 920 tandem arrays, 221 524 tandem duplication genes (Supplementary Table S5).

We also annotated a total of 1.83 million protein-coding genes of all genomes except for *A. thaliana*, with gene ontology terms (54) and KEGG orthology (55), and identify BLASTX (best hit) matches to *A. thaliana*. About 3.85 million functional elements were annotated, including InterPro domains (51), Pfam domains (53) (Supplementary Table S6). TPM values for 308 sets of RNA-seq data associated with 10 genomes, and variations of 1251 sets for re-sequencing data were integrated in BRAD. Through standardized calculation, the uniformity of data was greatly improved.

### Organization of features

**Figure 2.**
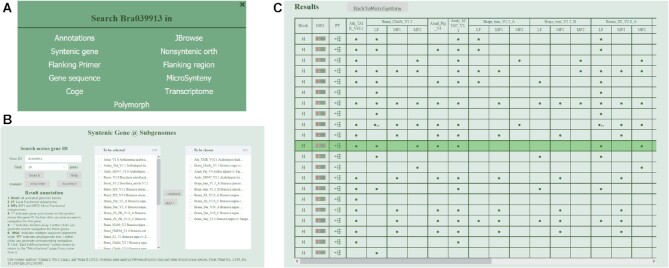
Search of Syntenic Gene @ Subgenome. (**A**) Pop-up retrieval system. (**B**) Search Syntenic genes in multiple genomes and subgenomes. (**C**) Result of syntenic genes.

*Pop-up retrieval system*. Multiple web pages formed the user interface and may be navigated between each other through a pop-up window (Figure 2A). Any gene ID within the database may be clicked to trigger the pop-up window, and then pages selected within this to navigate to and retrieve the gene. A range of relevant information may thus conveniently be retrieved using the pop-up retrieval system.

The major features of the user interface were organized into three menus, *Browse*, *Search* and *Tools*. In BRAD V3.0 the original *Browse* sub-menu was retained, apart from adding ‘Species info’ for a page to show the species where corresponding genomes has been collated. Most of the new features and updated features are located within the *Search* and *Tools* menus. Under *Search*, the original ‘Flanking region’ and ‘Synteny @ Genome’ (‘Syntenic Figure’ in BRAD V2.0) capability were retained. Minor changes were made to improve data retrieval and presentation for the ‘Annotations’, ‘Nonsyntenic Orth’ and ‘Gene sequence’. Substantial changes were made for ‘Syntenic gene @ Subgenome’ (‘Syntenic genes’ in BRAD V2.0). New features ‘Transcriptome’ and ‘Polymorph’ were also added in this menu. Under the *Tools*, the BLAST and genome browser were retained. Moreover, new tools were added, including ‘Multiple sequence alignment (MSA)’, ‘Gene Phylogeny’, ‘Genomic sequence retrieval’, ‘MicroSynteny’ and ‘Primer Design’.

Major updates and new features in the menus of *Search* and *Tools* are highlighted below.

### Search

#### Syntenic Gene @ Subgenome

This is a redesigned syntenic gene retrieval system across multiple reference genomes. By adding or removing genomes and subgenomes one by one to the ‘To be shown’ selection, users are able to order these as required (Figure 2B). We retained the same table style to indicate which syntenic genes were retrieved. In addition, two buttons were added to the table to allow ‘Multiple sequence alignment’ and ‘Gene Phylogeny’ to be readily displayed for the retrieved genes (Figure 2C).

#### Polymorph

**Figure 3.**
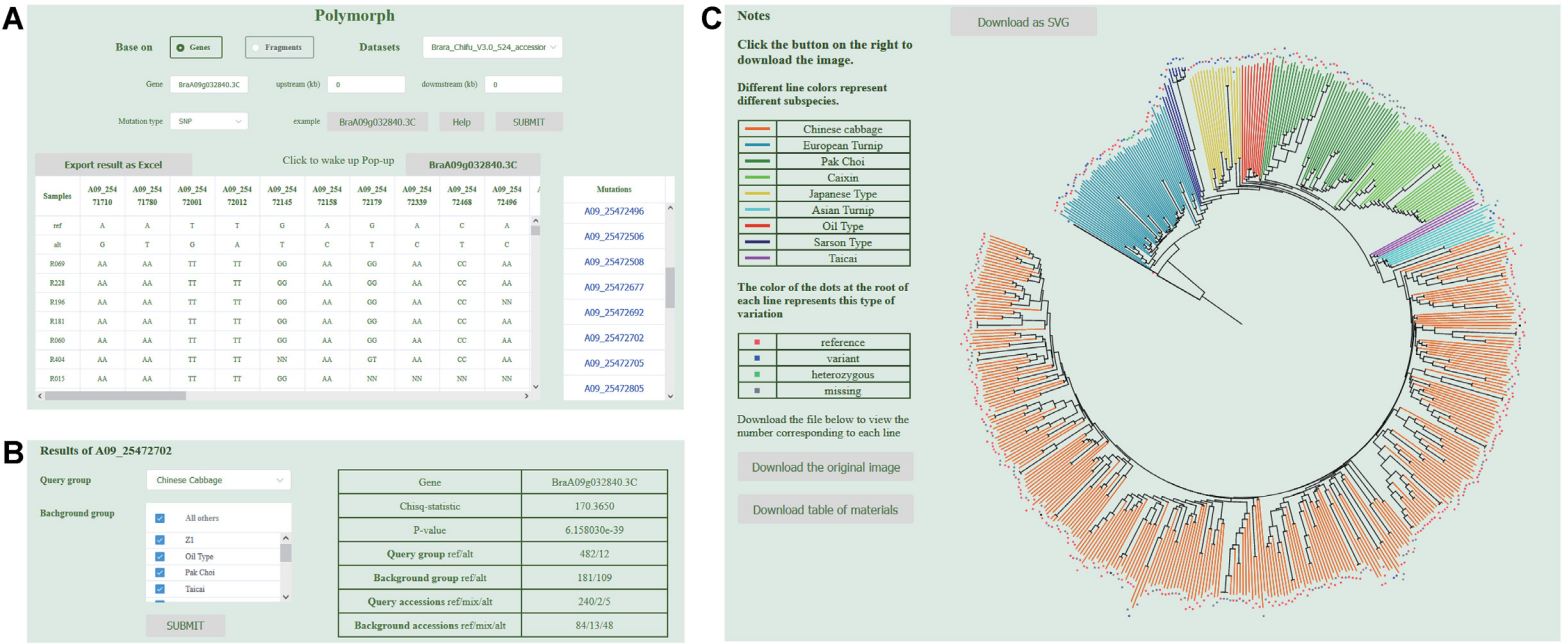
Variation data retrieval. (**A**) Variation retrieval and dynamic table display. (**B**) Variation distribution of different groups and the result of chi-square test. (**C**) Visualization of the distribution of variation in different groups.

This is a new module developed to make use of the increasing availability of genomic variation data. Across the 5 data sets, there are a total of ∼2.25M SNPs (from 199 samples against Chiifu V3.0) (42). For the *B. rapa* sets, there are 3.97M (524 on Chiifu V3.0) (41). For *B. oleracea* 3.85M (119 on JZS V1.1) (42), and 6.13M (119 on JZS V2.0). For *B. napus* 2.05M SNP (608 on Darmor-bzh V5) (43), respectively. Variation for each sample is displayed in the table and may be downloaded as an .xlsx file (Figure 3A). ‘Polymorph’ is not only a tool to search for SNP variations, but more importantly allows analysis of variation features among the genetic groups. We classified all genotype accessions into different groups based on either morphotypic features or whole genome phylogenetic branches. We added a function calculating P-value to help users better evaluate the role of variation among different groups. Users can optionally select query group and background group for query. The branches with different colour represent different groups, and the dots at the end of each branch represent its genotype (Figure 3C). The phylogenetic tree may be downloaded as an SVG file, which is very helpful for the users to see the distribution of the variation. Taking the variation site A09 25472702 in gene BraA09g032840.3C as an example, we selected Chinese cabbage as a query and all other groups as background. The P-value of 2.515576e–37 between query and background, indicated the variation site is strongly selected in Chinese cabbage (*B. rapa* subsp. *pekinensis*) (Figure 3B). Based on this module, we identified some unique variation sites in Chinese cabbage (Supplementary Table S7).

#### Other updates

**Figure 4.**
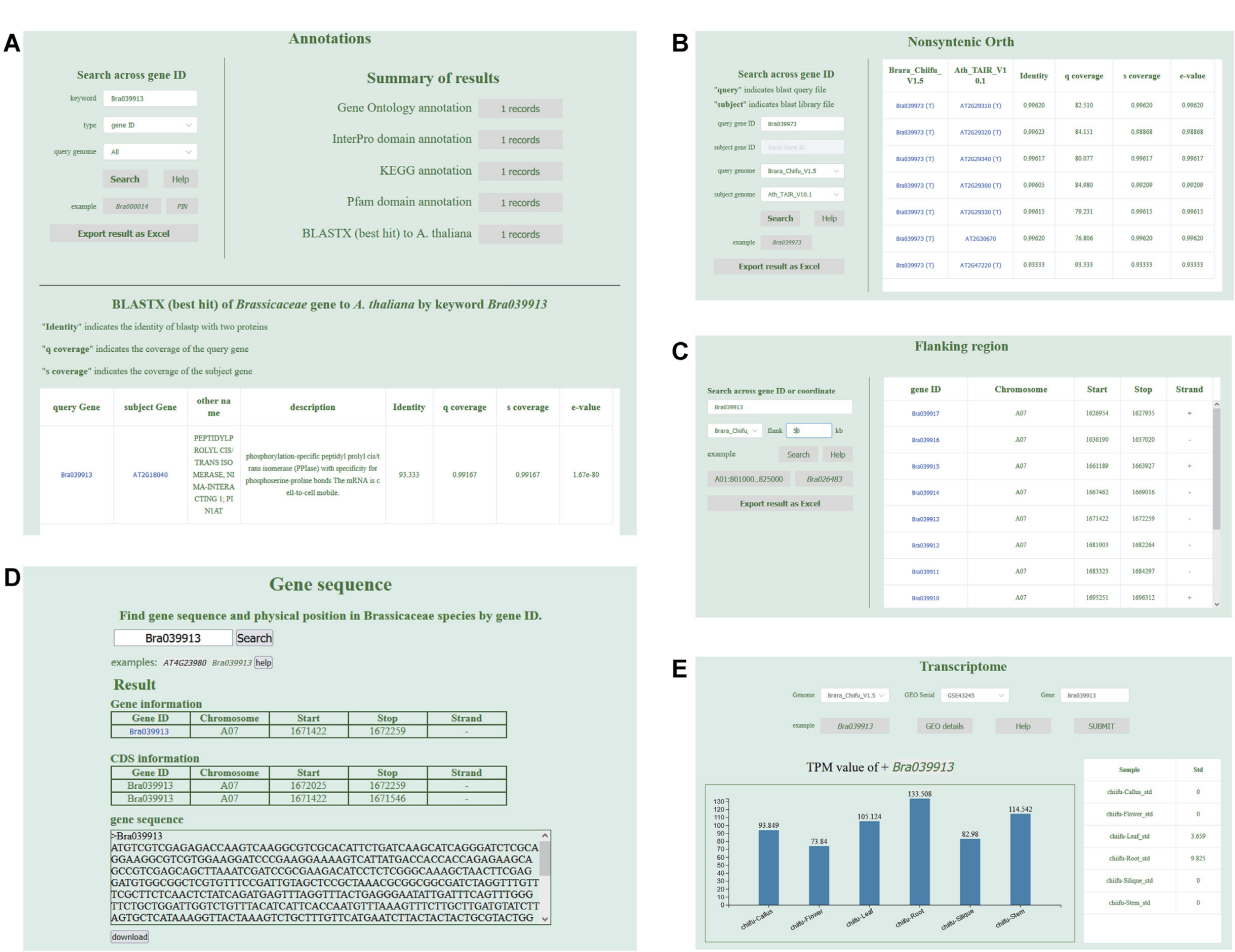
Several modules of BRAD. (**A**) Gene annotation information retrieval. (**B**) Non-syntenic ortholog genes search module. (**C**) Table showing the genes in flanking region. (**D**) Summary of gene sequence information. (**E**) Bar plot and table showing the gene expression level in TPM.

The Swiss-Prot and TrEMBL annotations were replaced with Pfam domain annotations. The annotation ID in the result set links to GO, InterPro, KEGG and Pfam databases to query the corresponding detailed information. ‘BLASTX to *A. thaliana*’ links the target gene to the model plant *A. thaliana* (Figure 4A). There are four genomes (*A. thaliana* TAIR V10.1, *B. rapa* cv. Chiifu V1.5, *B. rapa* cv. Chiifu V3.0, *B. oleracea* cv. JZS V2.0) that could be used as the subject genome of BLASTP to retrieve non-syntenic orthologous genes (Figure 4B), whereas BRAD V2.0 only had *Arabidopsis thaliana* V10.1. By entering the gene ID or coordinate and selecting the flank length, the genes existing in the region would be identified and shown in the table (Figure 4C). Location information and full-length sequence, CDS and protein sequence of the gene may be searched by gene ID (Figure 4D). The expression values of a gene in different tissues may be shown in images and tables (Figure 4E).

### Tools

#### MicroSynteny

**Figure 5.**
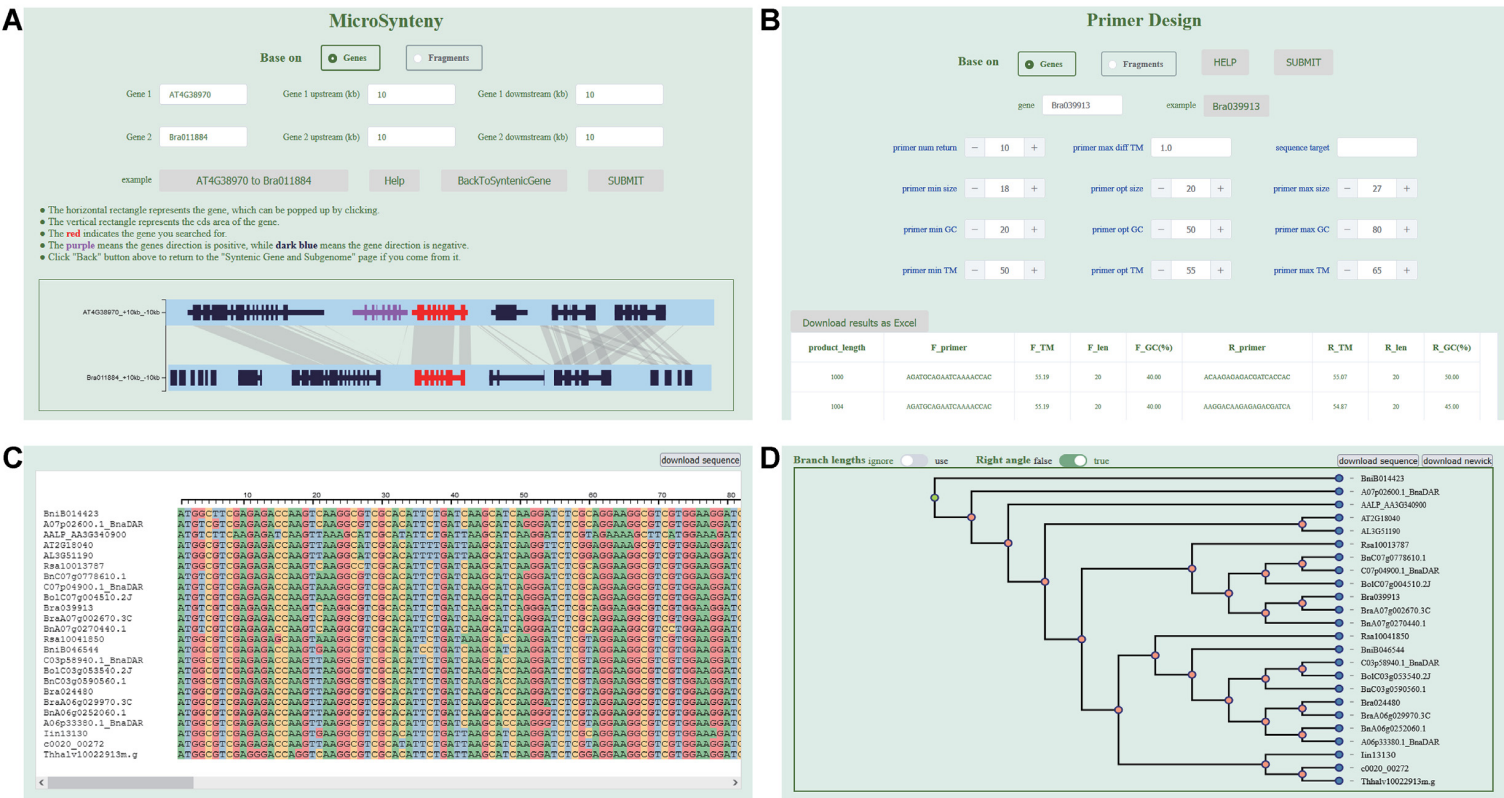
Some tools of BRAD. (**A**) Graphically show synteny between genomic region pairs. (**B**) Primers were designed to amplify the entire gene. (**C**) Multiple sequence alignment of syntenic genes. (**D**) Phylogenetic tree of syntenic genes.

This feature complements the ‘Syntenic Gene @ Subgenome’, and visualizes additional syntenic details between two genomic genes or regions. Both the gene ID and the genome region can be used as input in this module, and homologous regions between two segments are indicated in (Figure 5A). In addition, corresponding links were made through the pop-up retrieval system in ‘Syntenic Gene @ Subgenome’, so that the regional synteny between syntenic gene fragments may be queried conveniently. The images are able to be saved in SVG, PNG and JPG formats by right-clicking, and also expanded horizontally by scrolling up and down. Each horizontal rectangle may be clicked to trigger the pop-up window. The homologous fragments between the two fragments can also be further clicked to see the result of sequence alignment.

#### Primer design

This module was provided to design primers within selected genes (Figure 5B). When the user navigates from the pop-up window to this feature, the gene is presented as the default input, and a 150 bp flanking region is used to design the primer that would amplify the full length of the gene. All parameters are set with hyperlinks to the Primer3 manual (http://primer3.org/manual.html). In addition, a sequence can be input for primer design by selecting the button ‘Fragment’.

#### Multiple sequence alignment (MSA) and Gene Phylogeny (PT)

These are two new features provided to display the evolutionary relationship between protein and CDS sequences, which will help users further interpret the target gene. The second and third columns of syntenic gene results represent MSA and PT of all genes in each row (Figure 5C, D). Users are also free to add or subtract genes for queries.

#### Genomic sequence retrieval

The corresponding genome sequence may be obtained by submitting the information of reference genome, chromosome, start and stop position and strand, and downloaded as a .fasta format file.

#### BLAST

We integrated all the collected genome sequences, CDS sequences and protein sequences into Web BLAST so that users may perform BLAST retrieval of target sequence. The output may be downloaded in three different formats, HTML, TSV and XML.

#### Genome browser

We have implemented a genome browser using JBrowse together with plugins (63) to visualize genome sequences, gene models, markers, variations and omics data. JBrowse is more suited to visualizing multiple omics data than the GBrowse 2.0 (69) used by BRAD V2.0. Users may conveniently and efficiently access a region or gene of interest either by entering location or by selecting the ‘JBrowse’ option in the pop-up window. The tracks of reference sequence and gene models are embedded within this module.

## DISCUSSION

We updated BRAD by rebuilding the whole system with the MVVM framework and including new genomic data. In the updated BRAD, the synteny analysis system incorporates tools to analyse synteny at different levels, including genome, genomic region, gene and sequence levels. The provision of a wide range of synteny analysis tools will not only help genome comparison, but also help in unravelling gene function and in molecular marker design. We provided ‘Syntenic Gene @ Subgenome’ as a central module, so that users are able to make queries by navigating through the pop-up window to ‘MicroSynteny’, ‘Multiple sequence alignment’ and ‘Gene Phylogeny’ conveniently. ‘MicroSynteny’ allows users to view detailed homology alignments between genomic segments where syntenic genes are located. The ‘Multiple sequence alignment’ and ‘Gene Phylogeny’ tools help users better explore and understand the evolutionary relationships between syntenic genes. Moreover, within the ‘Syntenic Gene @ Subgenome’ we added two windows for selection and removal of genomes. This optimization provides considerable flexibility for organizing result output.

‘Polymorph’ is designed not only for showing and retrieving genome variation data, but also mining these data. For generating the genome variation data, we allowed genotype accessions to be organised into groups based on phylogenetic tree and morphotypes. For any query group, we provided a test of P-value for the distribution of the SNP in the query and the selected background. Polymorph represents a powerful tool for users to relate variation data to important developmental or agronomic traits.

Multiple new types of Brassicaceae genomic data, such as Hi-C, ChIP-seq, ATAC-seq, DAP-seq, methylation, single cell transcriptome etc., have been released over the past five years. However, a comprehensive database to integrate multiomics data for this important taxon has been lacking. The *Arabidopsis* eFP browser is an excellent tool for displaying gene expression profile data (70). With the accumulation of well-organized transcriptome experiments, especially in the extensively investigated *Brassica* crops, building an eFP browser in BRAD will increase the value of the database. Pan-genomes have been constructed for all *Brassica* species (71) and *Raphunas sativus* (72). Several genomes of *B. oleracea* have also been reported. Designing tools to analyse and visualize the pan-genome data is obviously an urgent need for the *Brassica* research community, and BRAD will continue to advance this effort.

## DATA AVAILABILITY

The BRAD can be accessed through the web server at http://brassicadb.cn. Transcriptome data processing and some of the backend code BRAD uses is available on GitHub repository at https://github.com/dahaigui/BRAD-scrpit.

## Supplementary Material

gkab1057_Supplemental_Files

## Notes

Present address: Xiaoning He, Institute of Plant Sciences Paris of Saclay (IPS2), UMR 9213/UMR1403, CNRS, INRA, Université Paris-Sud, Université d’Evry, Université Paris-Diderot, Sorbonne Paris-Cité, Bâtiment 630, 91405 Orsay, France.
