# Supplementary material for: BRAD V3.0: an upgraded Brassicaceae database
Source: Nucleic Acids Res. 2021 Nov 10;50(D1):D1432–41. doi: 10.1093/nar/gkab1057 (PMC8728314; doi:10.1093/nar/gkab1057)
Supplement: gkab1057_Supplemental_Files [file gkab1057_supplemental_files.zip › Supplementary Data.pdf]

SUPPLEMENTARY DATA

The Supplementary Data includes:

1. Supplementary Figure S1;
2. Supplementary Figure S2;
3. 7 Supplementary Tables in .xlsx format with one or more sheets.

Supplementary Table 1. List of programs and packages.

Supplementary Table 2. Species and genome assemblies integrated in BRAD.

Supplementary Table 3. RNA-seq data sources.

Supplementary Table 4. Distribution of syntenic genes.

Supplementary Table 5. The syntenic genes in the subgenome of triploidized genomes.

Supplementary Table 6. Distribution of tandem repeat genes.

Supplementary Table 7. Some variation in *Brassica rapa*.

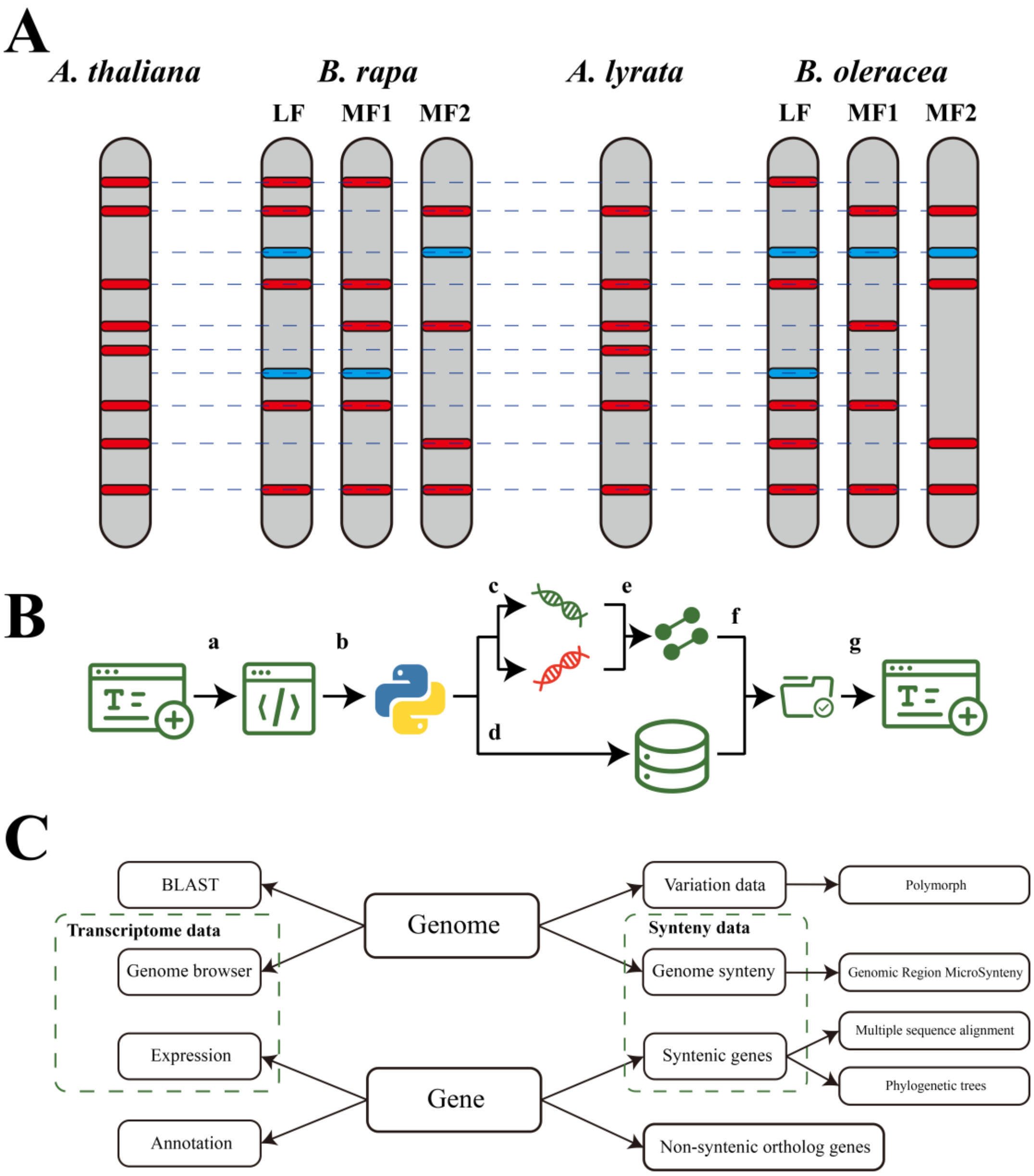

Supplementary Figure S1. **Syntenic gene table and MicroSynteny tools pipelines.** (**A**) Red represents pairwise syntenic gene with *A. thaliana*, blue represents non-syntenic gene pair with *A. thaliana*, but pairwise syntenic gene with *B. rapa*. *A. lyrata* represents species that have not undergone *Br-α* WGT event, *B. oleracea* represents species with *Br-α* WGT event. (**B**) Steps for implementing MicroSynteny. **a**) Data is transferred from the front end to the back end. **b**) The back end calls the Python script through Django. **c**) Two genome sequences were intercepted. **d**) The database was searched for gene and CDS information in the two fragment regions. **e**) Establish BLAST nucleic acid database with the second sequence as subject, and conduct default parameters alignment of blastn with the first sequence as query. **f**) The results obtained by e are filtered out for comparison shorter than 30bp, and packaged together with the retrieval results in the database, they are sent to the front end. **g**) Render the data using D3.js. (**C**) The relationship between the data sets and the relationship between the main functions.

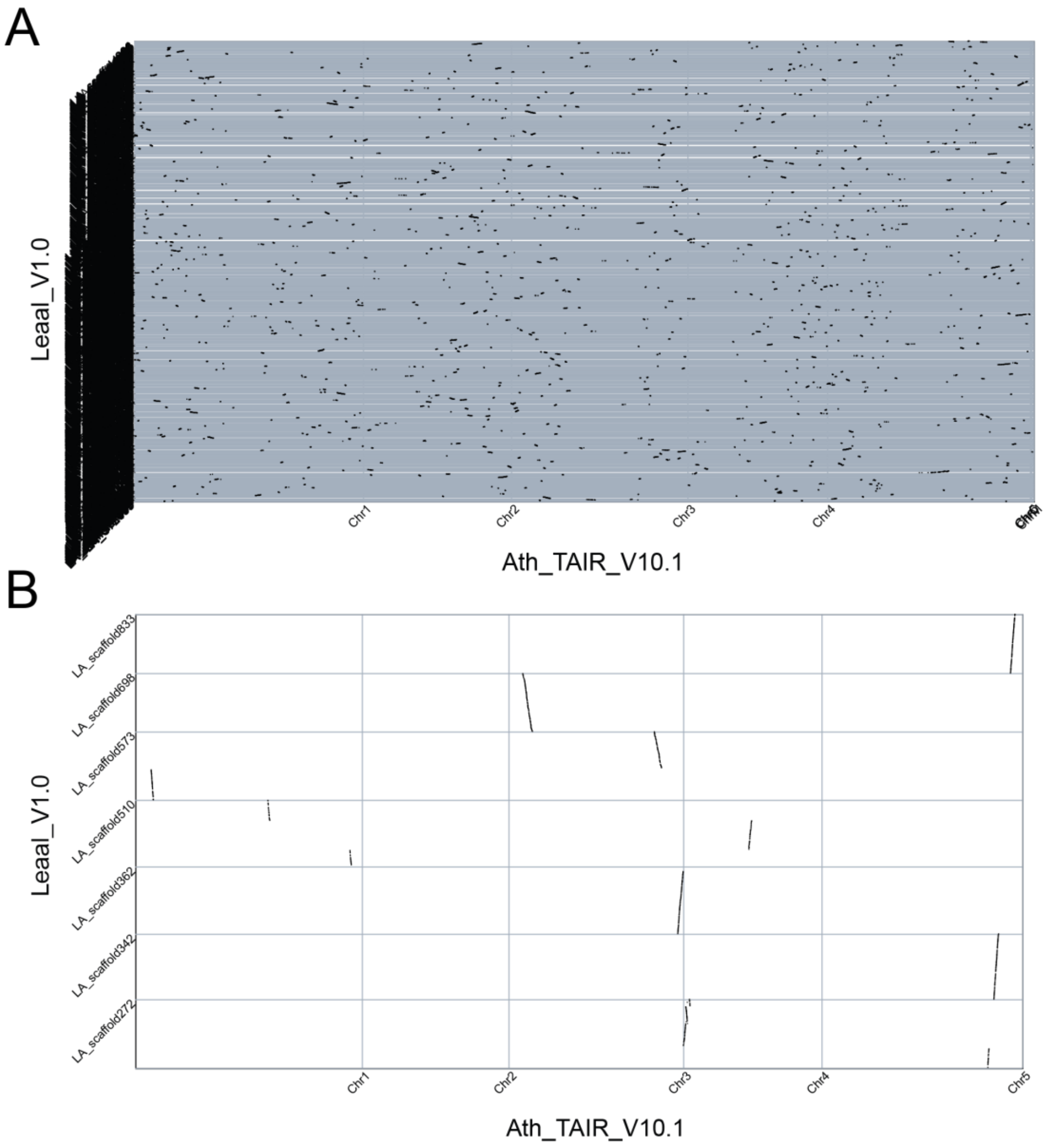

Supplementary Figure S2. **Synteny dotplot figure between *Leavenworthia alabamica* and *A. thaliana*. (A)** The result with no filter condition set. **(B)** The result of filtering out contig with fewer than 100 genes. It is obvious that the assembly quality of the *Leavenworthia alabamica* is poor
